# Significance of Heterogeneous Twist2 Expression in Human Breast Cancers

**DOI:** 10.1371/journal.pone.0048178

**Published:** 2012-10-25

**Authors:** Yubin Mao, Nini Zhang, Jinfei Xu, Zhijie Ding, Rongrong Zong, Zuguo Liu

**Affiliations:** 1 Department of Pathophysiology in Basic Science, Medical College of Xiamen University, Xiamen, Fujian, China; 2 Department of Surgery, Zhongshan Hospital of Xiamen University, Xiamen, Fujian, China; 3 Eye Institute and Xiamen Eye Center, Medical College of Xiamen University, Xiamen, Fujian, China; University of Nebraska Medical Center, United States of America

## Abstract

**Background:**

Twist2 (Dermo1) has been shown to mediate the epithelial-mesenchymal transition (EMT) to promote tumor invasion and even metastasis. However, the involvement of EMT in breast cancer progression is highly debated, partially due to clinical observations showing that the majority of human breast carcinoma metastases express E-cadherin and maintain their epithelial morphology. The molecular mechanism by which Twist2 participates in EMT of breast cancer in vivo remains poorly understood.

**Methods:**

We examined Twist2 expression pattern in human breast carcinomas by western blot and tissue microarray, and analyzed Twist2 cellular localization by confocal microscopy, cell fractionation and other approaches.

**Results:**

Twist2 expression was significantly increased in breast cancer. Cytoplasmic Twist2 positive cancer cells expressing E-cadherin on the cellular membrane were mainly located at tumor center of primary carcinomas and lymph metastases, while cancer cells with nuclear Twist2 clearly showed loss of E-cadherin and were detected at the invasive front in ductal breast carcinomas. In addition, ectopically stable-expressed Twist2 was found to localize in the cytoplasm of cancer cells. Collectively, these data indicate that upregulation of cytoplasmic Twist2 is correlated with tumor histological type and tumor metastasis in human breast cancers.

**Conclusion:**

The differential cellular distribution of Twist2 may be associated with tumor progression. The cytoplasmic Twist2 in cancer cells at tumor center of primary carcinomas and lymph metastases contributes to the maintenance of epithelial cancer characteristics expressing E-cadherin in a noninvasive state, while the nuclear Twist2 at the cancer invasion front activates EMT to deprive epithelial property of neoplastic cells, thus facilitating invasion and metastasis. These findings suggest that heterogeneous expression of Twist2 in tumors may have a functional link to tumor progression.

## Introduction

Epithelial-mesenchymal transition (EMT) has been implicated as a means by which normal or transformed epithelial cells acquire the abilities to invade, resist apoptosis, and disseminate during development and cancer progression [1], [2], [3]. EMT, although not always the case, is generally considered as a prerequisite step during initial phase of metastasis. Multiple transcriptional factors, including Twist, Snail, and Slug, orchestrate the EMT and the migratory processes during embryogenesis. These factors have also been shown to promote cancer invasion and in metastasis in many experimental models of malignant tumors [4], [5], [6]. Growing evidence suggests that these transcription factors may regulate each other and control overlapping sets of target genes. The molecular mechanisms underlying the regulation of their interactions and expressions have not been well defined [7], [8].

Recent understanding on EMT largely came from in vitro studies [9], [10]. It's difficult to validate whether carcinoma cells in human primary tumors have gone through an EMT in vivo. It is well-known that cells undergoing an EMT not only change their cellular characteristics to acquire motility and invasiveness but also develop new interactions with the extracellular environment. A hallmark of EMT is the loss of E-cadherin expression. However, some clinical observations showed that the majority of human breast carcinoma metastases express E-cadherin and maintain their epithelial morphology, suggesting that they have disseminated without switching to a mesenchymal phenotype or undergone mesenchymal-epithelial transition (MET) after metastatic growth [11], [12].

Twist1 and Twist2 (dermo1), the basic helix-loop-helix (bHLH) transcriptional factor family, share more than 90% sequence homology and structural similarity at bHLH and C-teminal domains. They also overlap in temporal and spatial expression, and play critical roles in embryonic mesenchymal development [13]. A number of studies showed the important role of Twist1 in promoting cell survival, cell invasion and immigration [14], [15], and facilitating tumor angiogenesis [16]. Both Twist1 and Twist2 are known to mediate EMT in human cancers [17]. Twist1 is a key regulator of metastasis. It has been shown that Twist1 promotes EMT through down-regulation of E-cadherin in subsets of sporadic invasive human lobular breast cancer [18], but little is known about the expression pattern of Twist2 [19], [20]. Twist2 activates EMT programs and facilitates a cancer stem cell phenotype in breast cancer [19]. However, the role of Twist2 in promoting breast cancer invasion and metastasis has not been established in the context of the breast microenvironment. In addition, the identification and clinical relevance of Twist2 in breast cancer is not known.

We showed that Twist2 was up-regulated in human primary breast carcinoma tissues compared with the matched normal breast tissues. Twist2 was expressed mostly in cytoplasm as demonstrated by immunohistochemical (IHC) assay in tissue microarray. Cytoplasmic Twist2 was associated with tumor histological type, the TNM clinical stage and tumor metastasis. Our study showed that, in some cases of invasive ductal breast carcinoma, Twist2 were mainly localized in cytoplasm of cancer cells expressing E-cadherin at tumor center and the lymph metastases. In contrast, nuclear Twist2 were detected in cancer cells located at the invasive margins of primary breast cancer. But the nuclear Twist2 positive cells surrounding the lymph metastases showed loss of E-cadherin at the tumor invasion fronts. In this study we report that Twist2 promotes breast cancer invasion through loss of E-cadherin. Our data suggested that there was a link between nuclear Twist2 and EMT and the EMT process depends on Twist2 cellular location. These results demonstrate an important role of Twist2 in breast cancer invasion and indicate that Twist2 may be a new EMT indicator for dissemination of breast cancer.

## Results

### Twist2 is up-regulated in breast cancer

**Figure 1:**
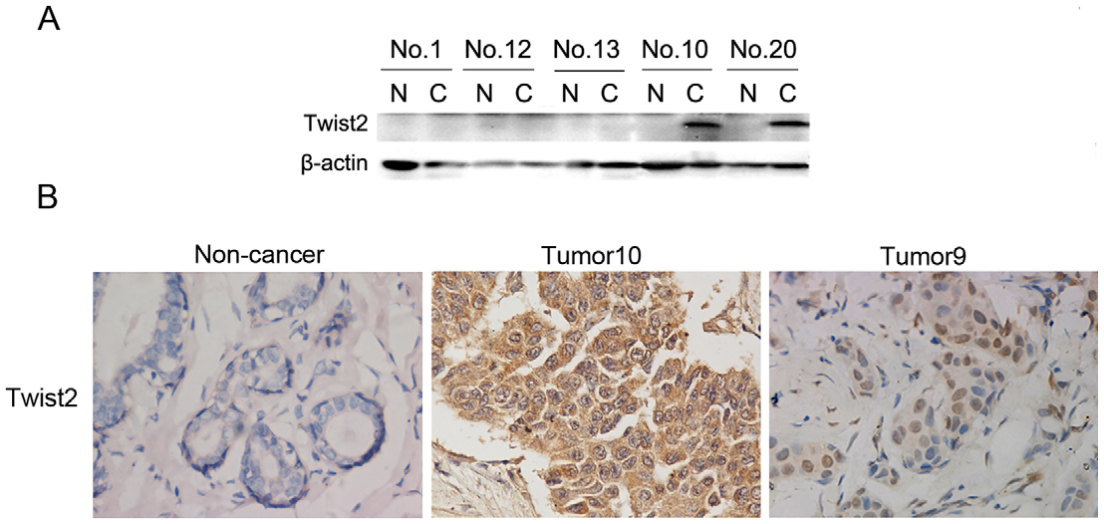
Twist2 is up-regulated in breast cancer. **A.** Immuboblot analysis of Twist2 expression in fresh breast cancer tissues. Twist2 was up-regulated in breast carcinomas relative to matched normal breast tissues. **B.** Immunohistochemical (IHC) staining of Twist2 on sections of paraffin-embedded breast carcinomas and normal breast tissues. Twist2 was localized in the cytoplasm (Tumor 10) or nucleus (Tumor 9), depending on the tumor specimen (magnification:×400).

To determine Twist2 expression in breast cancers, we initially performed Western blot in human breast carcinoma samples and matched normal breast tissues. We collected 25 cases of ductal breast carcinoma, 4 cases of lobular carcinoma, 3 cases of mixed breast cancer, and 7 normal breast tissues without breast disease from Zhongshan Hospital of Medical College of Xiamen University. In these fresh breast cancer tissues, Twist2 showed up-regulation in breast carcinomas as demonstrated by western blot analysis (Figure 1A). We then analyzed the expression and location of Twist2 on the matched breast cancer tissues and normal tissues by immunohistochemical staining. No positive expression of Twist2 was detected in 7 normal breast samples or adjacent normal breast tissues in 32 breast cancer patients. In contrast, Twist2 was detected in breast carcinoma tissues (Figure 1B). These results indicate up-regulation of Twist2 in these breast carcinomas.

### Pathological characteristics of Twist2-expressing breast carcinomas

Several studies on function of Twist1 in breast cancer have identified an inverse correlation between Twist1 and E-cadherin expression in invasive lobular carcinomas (ILC) [5]. These cells underwent the molecular and morphological features associated with an EMT which included the loss of expression of E-cadherin. However, little is known on the functional significance of Twist2 expression in breast cancer. To determine the expression pattern of Twist2 in breast carcinomas, we performed immunohistochemical staining of Twist2 on a tissue microarray that includes 7 cases of normal breast tissue, 6 cases of fibroadenoma, 13 cases of mammaryadenosis, 16 cases of mastitis, and 141 cases of breast cancer. Our results showed that there is no expression of Twist2 in normal breast tissues (0%, 0 of 7 cases), weak expression of Twist2 in benign breast diseases including mammaryadenosis, fibroadenosis, and mastitis (25%, 7 of 28 cases), and moderate to strong expression of Twist2 in malignant tumor (73.94%, 105 of 141 cases). This data indicated Twist2 expression was preferentially increased in breast cancer. Twist2 is overexpressed in a majority of breast carcinomas. The positive rates are as following:74.78% ductal carcinomas (86 of 115 cases),68.18% lobular carcinomas (15 of 22 cases), and 100% squamous-cell carcinomas (4 of 4 cases) (Table 1). Twist2 expression also increased significantly with the TNM clinical stage classification as following:50% stage 0 (11 of 22 cases), 68.49% stage I/II (50 of 73 cases), 95.65% stage III/IV (44 of 46 cases) (Table 1). Twist2 expression is upregulated significantly with tumor metastasis, especially distant lymph nodes metastasis (100%, 31 in 31, Table 1). In addition, there is no correlation between the Twist2 expression and the age of patients.

**Table 1:** Clinical pathological characteristics of Twist2-associated breast carcinomas.

	Cases	Twist2				
		−	+	*X^2^*	*P*	
Tissue type				46.83	<0.05	
Normal breast tissues	7	7	0			
Fibroadenoma	6	5	1			
Mammaryadenosis	13	11	2			
Mastitis	16	12	4			
Malignant tumor	141	36	105			
Tumor histological type				1.84	>0.05	
Ductal	115	29	86			
Lobular	22	7	15			
Squamous cell	4	0	4			
TNM clinical stage				19.16	<0.05	
0	22	11	11			
I/II	73	23	50			
III/IV	46	2	44			
Metastasis				13.69	<0.05	
Non-metastasis	90	29	61			
Regional lymph nodes metastasis	20	7	13			
Distant lymph nodesmetastasis	31	0	31			
Age(years)				0.05	>0.05	
≥50	61	15	46			
<50	80	21	59			

TNM clinical stage of breast cancer is according to the American Joint Committee on Cancer (AJCC) TNM system.

In addition, we detected a heterogeneous expression pattern of Twist2 within breast cancers. Twist2 was detected in 105/141 cases (Table 2). In these breast carcinoma specimens, Twist2 was more often detected in the cytoplasm only (50/105, 48%) or both the cytoplasm and nucleus (38/105, 36%) compared to the nucleus only (17/105, 16%). Interestingly, presence of Twist2 in the cytoplasm only correlated with tumor histological type, mainly in ductal carcinomas of breast (45/115, 39.13%) relative to lobular carcinomas (2/22, 9.09%). Presence of Twist2 in the nucleus only also correlated with tumor histological type, mainly in lobular carcinomas of breast (8/22, 36.36%) relative to ductal carcinomas (8/115, 6.96%). Intracytoplasmic Twist2 increased with tumor metastasis, while Twist2 expression in nucleus had no correlation with TNM clinical stage or tumor metastasis (Table 2). Collectively, our results indicated that Twist2 was strongly expressed in malignant breast cancer. Twist2 is mainly localized in the cytoplasm of ductal breast carcinomas, and correlated with tumor histological type and tumor metastasis. Twist2 might be a new candidate for tumor metastasis of breast cancer.

**Table 2:** The expression of Twist2 in cytoplasm and nucleus of breast carcinomas.

	Cases	Cytoplasm only			
		−	+	*X^2^*	*P*
Tumor histological type				10.61	<0.05
Ductal	115	70	45		
Lobular	22	20	2		
Squamous cell	4	1	3		
TNM clinical stage				5.13	>0.05
0	22	17	5		
I/II	73	50	23		
III/IV	46	24	22		
Metastasis				9.75	<0.05
Non-metastatic tumor	90	62	28		
Regional lymph nodes metastasis	20	16	4		
Distant lymph nodes metastasis	31	13	18		

	Cases	Nucleus only			
		−	+	*X^2^*	*P*
Tumor histological type				13.44	<0.05
Ductal	115	107	8		
Lobular	22	14	8		
Squamous cell	4	3	1		
TNM clinical stage				5.55	>0.05
0	22	22	0		
I/II	73	65	8		
III/IV	46	37	9		
Metastasis				2.82	>0.05
Non-metastatic tumor	90	82	8		
Regional lymph nodes metastasis	20	17	3		
Distant lymph nodes metastasis	31	25	6		

	Cases	Both cytoplasmic and nuclear expression			
		−	+	*X^2^*	*P*
Tumor histological type				1.26	>0.05
Ductal	115	82	33		
Lobular	22	17	5		
Squamous cell	4	4	0		
TNM clinical stage				0.07	>0.05
0	22	16	6		
I/II	73	54	19		
III/IV	46	33	13		
Metastasis				0.43	>0.05
Non-metastatic tumor	90	65	25		
Regional lymph nodes metastasis	20	14	6		
Distant lymph nodes metastasis	31	24	7		

TNM clinical stage of breast cancer is according to the American Joint Committee on Cancer (AJCC) TNM system.

### Differential levels of Twist2 in tumor center and invasion front within a primary breast carcinoma and the adjacent lymph metastases

As E-cadherin is the key downstream target of EMT, we examined whether Twist2 histopathologic expression pattern is consistent with alteration of E-cadherin expression. A comparison of the E-cadherin and Twist2 expression was performed on the adjacent tumor sections of invasive ductal carcinoma (IDC) because this type of tumor accounts for 80% of breast cancers. The result did not show an inverse correlation between Twist2 and E-cadherin (Table 3). Being an important EMT inducers [21], Slug engenders breast cancer cells with stem cell-like properties and promotes metastases [22], [23]. We also analyzed Slug expression relative to Twist2 and E-cadherin expression in IDCs and found no obvious correlation among them (Table 3).

**Table 3:** Spearman's correlation between the immunostaining of Twist2, E-cadherin and Slug.

Marker	Correlation	Twist2	E-cadherin	Slug
Twist2	Correlation coefficient	1.000		
	P value	0.000		
	N	71		
E-cadherin	Correlation coefficient	0.217	1.000	
	P value	0.267	0.000	
	N	28	28	
Slug	Correlation coefficient	0.434	−0.034	1.000
	P value	0.056	0.888	0.000
	N	20	20	20

Spearman's rank correlation was used to determine whether there was a positive or negative correlation.

**Figure 2:**
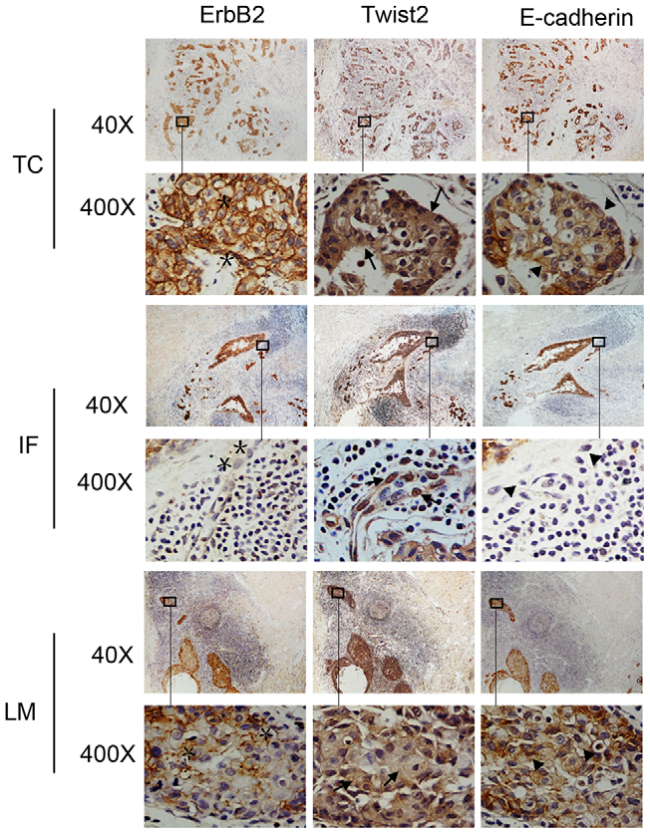
The expression patterns of ErbB2, Twist2 and E-cadherin in human breast carcinomas. Representative images ErbB2, Twist2 and E-cadherin IHC staining in tumor central areas (TC, first and second row) and invasive front (IF, third and fourthrow) of the primary tumor, and surrounding lymph metastasis (LM, fifth and sixth row) are shown. Boxes indicate magnified regions in stained serial sections. Specific positive staining is shown in brown color, and nuclei were counterstained in blue. Tumor cells are clearly polarized in the differentiated gland-like or tubular structures areas in both primary tumor center and metastases. A high ErbB2 indicating strong proliferation (star) was detected only in differentiated area of *TC* and *LM*. Loss of gland-like growth tumor cells did not express ErbB2 in the corresponding IF. Twist2 is only detectable in the cytoplasm (arrows) in TC and LM. In contrast, tumor cells at the invasive front lose their polar orientation and display fibroblast-like shape. This morphological change is accompanied by nuclear accumulation of Twist2 (arrows). Consistently, tumor cells in differentiated areas of the primary tumor and in metastases express E-cadherin on cell membrane (arrowheads). Disseminating tumor cells at the invasive fronts with nuclear Twist2 completely lost E-cadherin (arrowheads).

**Figure 3:**
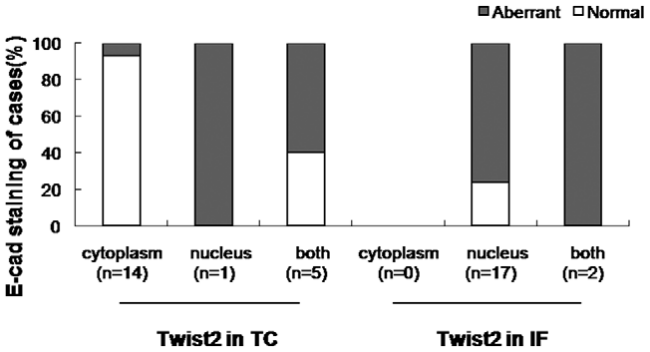
The association between subcellular localization of Twist2 and expression of E-cadherin in cells of the tumor center (TC) and invasive front (IF). Twist2 in the nucleus was related to the loss of E-cadherin expression. Aberrant E-cadherin expression was defined as weak or loss of expression, normal was defined as normal level and subcellular localization of E-cadherin. Cytoplasm indicated “Cytoplasm only”. Nucleus indicated “Nucleus only”.

Cancer invasion, characterized by a loss of epithelial differentiation, dissociation and migration of single cancer cells, is thought to happen only at the periphery (tumor invasive front) of the primary carcinomas. The cancer cells in the invasive fronts are known to have undergone an EMT trait, such as loss of E-cadherin [21]. When the tumor invasion front (IF) was compared with the tumor center (TC), a correlation can now be detected between the sub-cellular location of Twist2 and E-cadherin expression. Twist2 was only detectable in the cytoplasm of tumor cells at TC and the lymph metastases (LM). Those cells were clearly polarized in the differentiated gland-like or tubular structures areas in both primary tumor center and metastases (Figure 2). In addition, the cancer cells were marked with high ErbB2 which indicated strong proliferation of epithelial cancer cells. In contrast, tumor cells at IF lost their polar orientation and displayed fibroblast-like shape, and were negative for ErbB2 expression. This morphological change is accompanied by nuclear accumulation of Twist2. Consistently, tumor cells in the areas of TC and LM expressed E-cadherin on cell membrane (Figure 2). Disseminating tumor cells at IF with nuclear Twist2 completely lost E-cadherin. Notably, a significant amount of nuclear Twist2-expressing cells at the tumor invasion front (IF) showed loss of E-cadherin in those IDC samples with surrounding lymph metastasis (76.47%, 13 of 17 cases), while in TC of the same cases, cells with Twist2 in the cytoplasm only retained E-cadherin staining on membrane or cytoplasmic E-cadherin staining (92.86%, 13 of 14 cases, Figure 3). Since Twist2 is a member of b-HLH family transcription factors, it must be in nuclei to activate transcription of downstream signal molecules. Twist2 in the nucleus was related to the abnormal expression of E-cadherin in IF, while cytoplasm Twist2 was related to cytoplasm or membrane E-cadherin expression in TC. As Table 4 shows, there's a correlation between nuclear Twist2 and loss of E-cadherin. Thus, EMT at the IF, as evidenced by the loss of E-cadherin and fibroblastic morphology, correlated well with the presence of Twist2 in the nucleus. Cytoplasm Twist2 of cancer cells both in primary TC and the LM expressed E-cadherin and maintain their epithelial morphology, suggesting that the cancer cells in LM may have reacquired this morphology by a reversion of EMT (i.e. performing a mesenchymal to epithelial transition) [24].

**Table 4:** Spearman's correlation between the regulation of Twist2 and E-cadherin.

Marker	Correlation	E-cadherin
Twist2	Correlation coefficient	0.514^*^
	P value	0.020
	N	20

Spearman's rank correlation was used to determine whether there was a positive or negative correlation. Twist2 with “Nucleus only” was correlated with aberrant E-cadherin expression.

### Ectopic expression of nuclear Twist2 in MCF7 cells with down-regulates E-cadherin

**Figure 4:**
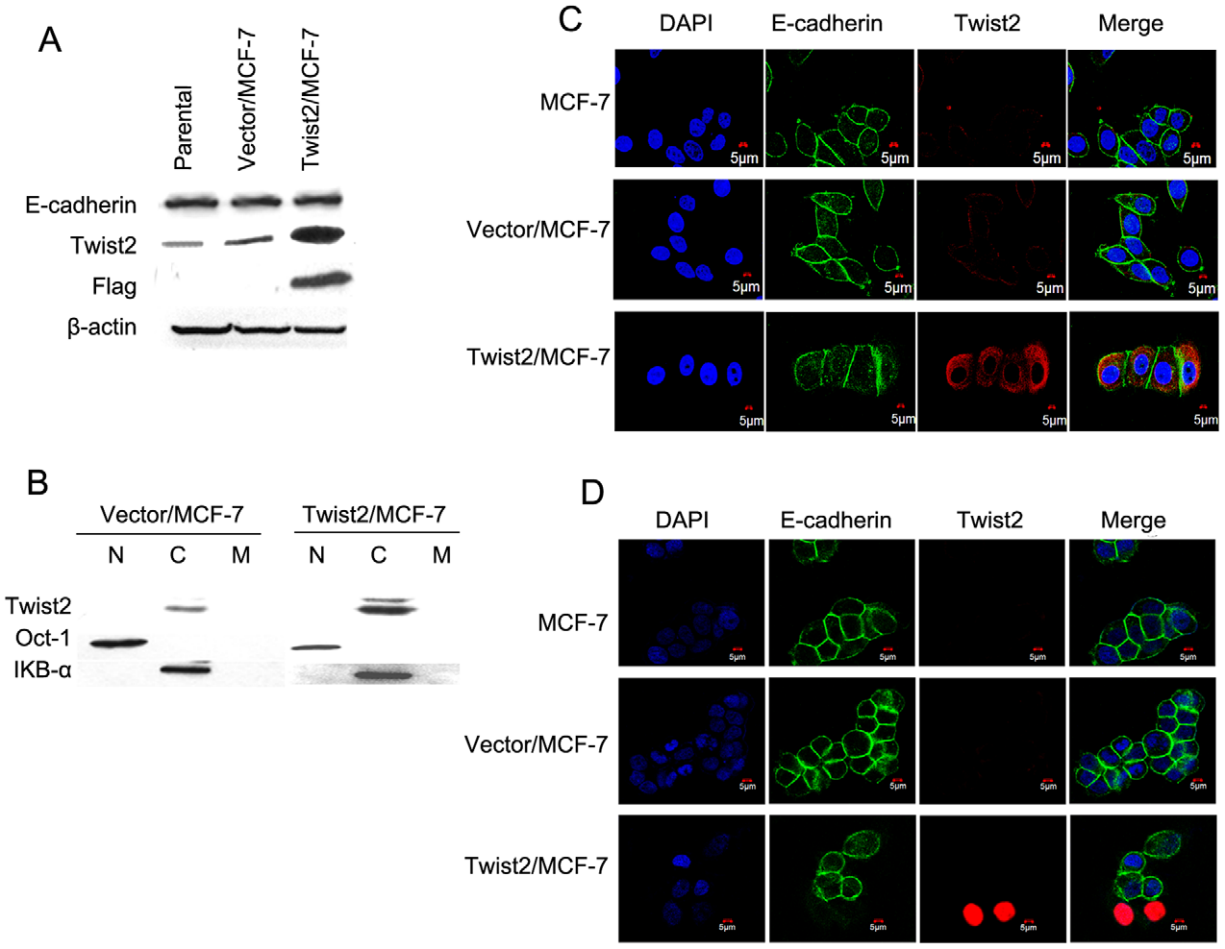
The regulation of E-cadherin expression by Twist2 in breast cancer cells. **A.** Immunoblot analysis showing that strong expression of E-cadherin was found with cytoplasic Twist2 expression. Stable expression of ectopic Twist2 in breast cancer cells (Twist2/MCF-7) were verified by western blot with anti- Twist2 and anti-flag antibodies. No obvious changes of E-cadherin was detected between Twist2/MCF-7, Vec/MCF-7 (the vector control), and the parental group. **B.** Immunoblot analysis of Twist2 in subcellular fractions showing that Twist2 was localized in the cytoplasm. Oct-1 indicated nuclear fraction and IkB-α indicated cytoplasmic fraction. The cells were from the stably transfected samples. **C.** Immunofluorescent staining of Twist2 and E-cadherin in MCF-7 cells showing cells with Twist2 (in red) in cytoplasm expressed E-cadherin (in green) on cell membrane. Nuclei were counterstained with DAPI (in blue). The cells were from the stably transfected samples. **D.** Immunofluorescent staining showing that transient over-expression of Twist2 (in red) in nuclei caused loss of E-cadherin in the same cancer cells. Cells without nuclear Twist2 retained expression of E-cadherin on membrane. Nuclei were counterstained with DAPI (in blue).

To further investigate the regulation of E-cadherin expression by Twist2, we established Twist2 exogenous over-expression in MCF7 cell line. In this epithelial tumor cell line, no obvious downregulation of E-cadherin was detected when Twist2 was stably over-expressed (Figure 4A). Subcellular fraction analysis and immunofluorescent staining showed that stably expressed Twist2 was located in cytoplasm of the cancer cells (Figure 4B, C). Most cells with cytoplasmic Twist2 mostly showed E-cadherin on cell membrane (Figure 4C), which is similar to the cancer cells at tumor center or metastases in vivo. In contrast, transiently expressed Twist2 was strongly detected in nuclei of cancer cells with loss of E-cadherin (Figure 4D). Little is known so far on how and when Twist2 translocates into nuclei. Here, we show that EMT program may be activated transiently through nuclear Twist2, but not cytoplasmic Twist2. Taken together, our results suggest that nuclear Twist2 may activate EMT transiently in the tumor invasion front, while cytoplasmic Twist2 contributes to the maintenance of epithelial cancer characteristics in tumor center or LM metastases in breast cancer.

## Discussion

It has been well recognized that EMT plays a critical role in cancer metastasis [1]. However, the difficulty to directly demonstrate the role of EMT in metastasis in vivo is to validate cancer cells that have undergone an EMT in primary human tumor specimens [8]. The molecular mechanism associated with the involvement of EMT in tumor metastasis is still highly debated. As clinical observations showed that the majority of human breast carcinoma cells in metastases express E-cadherin and maintain their epithelial morphology, cancer cells may have disseminated without switching to a mesenchymal phenotype [11], [12]. The master regulators of tumor invasion and metastasis were largely unknown [10]. Twist1 is one of essential factors to promote tumor metastasis [25]. The hypothesis that cancer cells routinely undergo a complete EMT program is likely to be simplistic. In breast cancer, Twist1 only partially induced an EMT program [26]. Twists belong to bHLH transcription factor family which form either homo-or-heterodimers with other bHLH proteins to bind to a core E-box (CANNTG) sequence on the promoter region of target genes such as E-cadherin [17]. It has been reported that Twist2 could activate EMT programs to facilitate a cancer stem cell phenotype in breast cancer recently [19]. But how Twist2 participates in EMT of breast cancer in vivo remains poorly understood [19]. The present data indicate that Twist2 staining was a reliable predictor in the prognosis of breast cancer patients (Table 1). Twist2 increased significantly with tumor metastasis, especially in cytoplasm of ductal carcinoma of breast cells (Table 2). Additionally, cytoplasm Twist2 expression was mainly in ductal carcinoma of breast relative to lobular carcinoma (39.13% vs. 9.09%). Twist1 has also been shown to be expressed in cytoplasm but not nucleus of human hepatocellular carcinoma (HCC), whereas E-cadherin was localized on membranes [27]. Twist2 overexpression was significantly linked to cervical cancer progression recently [28]. Therefore, these findings suggest that Twist2 plays a crucial role in the progression of breast carcinoma. Assessment of Twist2 expression status may provide clinically useful prognostic information in patients with breast cancer.

Arising from epithelial tissues progressed to higher pathological grades of malignancy, breast cancer cells typically developed alterations in morphology as well as in their attachment to the extracellular matrix. This EMT process is characterized by the down-regulation of the molecular markers of epithelial cells together with the loss of intercellular adhesion. Loss of E-cadherin expression is a hallmark of EMT. As far as we know, Twist2 and Slug are also EMT inducers [23]. High level of Slug is a determinant in the progression of metastatic breast cancer [22], [29]. Our results showed no correlation between Twist2, E-cadherin, and Slug (Table 3). Some of this controversy might be due to EMT being a transient, reversible process occurring during the course of tumor metastasis [2], [30]. In this reversible EMT model, carcinoma cells activate the EMT program to achieve local invasion and dissemination to distant organs. Once they have reached those organs, these mesenchymal cells may revert via an MET to an epithelial identity and thereby regain proliferative ability and the ability to form epithelial growths in distant organs or tissues.

In support of the transient EMT model, we have found differences between cells of the invasion front and cells of the tumor center in respect to both the subcellular localization of Twist2 and expression of E-cadherin (Table 4). In cells of the tumor center and lymph metastases, cytoplasmic expression of Twist2 correlated with an epithelial morphology and normal E-cadherin expression (Figures 2 and 3). In contrast, the fibroblast-like breast cancer cells at the invasion front were characterized by a nuclear localization of Twist2 and loss of E-cadherin (Figures 2 and 3). By forming adherent junctions with adjacent epithelial cells, E-cadherin may help to assemble epithelial cell sheets and maintain the quiescence of the cells within these sheets [2], [3].

Moreover, cancer cells at the invasive margins of certain carcinomas may have undergone an EMT, as these cancer cells are subjected to microenvironmental stimuli distinct from those received by cancer cells located in the cores of these lesions [31]. The cytoplasm Twist2 at tumor center and the lymph metastases is likely to contribute to the maintenance of epithelial cancer characteristics with E-cadherin expression in a noninvasive state, while the nuclear Twist2 activates EMT in the cancer invasion front, thereby deprive neoplastic epithelial cells with E-cadherin to facilitate metastasis (Figure 2). In addition, we found a significant positive correlation between nuclear Twist2 expression and loss of E-cadherin pattern (*P*<0.05, *r* = 0.762; n = 20, Table 4; Figure 3). In head and neck cancers, decrease of keratinization and loss of cellular cohesiveness were also observed in the invasive front of tumor tissue where cells underwent EMT with preserved membraneous E-cadherin in normal and tumor center [32]. Our findings suggest that heterogeneous expression of Twist2 in tumors may have different function: cytoplasm Twist2 might participate in some cellular events to maintain the epithelial cancer cell growth when residing in the primary tumor or metastasis, while Twist2 in nucleus is involved in EMT transiently.

This result was further confirmed by evaluating the Twist2 expression pattern and exogenous overexpression of Twist2 in breast cancer cells. Our results suggest that Twist2 is continuously localized in the cytoplasm of carcinoma cells that were stably selected, which may help carcinoma cells maintain the similar histological behavior in a noninvasive state. We need to further explore this possibility in the future. Cells with cytoplasm Twist2 showed no obvious change in cellular morphology with strong membranous or cytoplasm expression of E-cadherin in primary breast cancers or metastases. Only those transiently transfected cells with Twist2 overexpression in nuclei showed loss of E-cadherin. Triggered by some signal from the activated stroma during invasion, Twist2 could accumulate in nuclei during initial invasion and metastasis, and functions as a transcriptional factor to regulate EMT. Twist2 in nuclei could remarkably repress E-cadherin in the invasion edge to promote EMT, thus increase cell motility and invasiveness to enter the new adjacent tissue [1], [33].

Recent findings suggest that cells undergone EMT were responsible for degrading the surrounding matrix to enable invasion and intravasation of both EMT and non-EMT cells. Only those non-EMT cells that had entered the blood stream were able to re-establish colonies in the secondary sites [10]. Similarly, high nuclear β-catenin expression at the invasion front and less nuclear β-catenin in central tumor regions exist in colorectal carcinoma tissues [31]. Thus, carcinoma cells may experience EMT in invasive front area, then the MET (mesenchymal-epithelial transition) process in metastasis. When cancer cells move to their new homing sites, Twist2 redistributes to the cytoplasm with E-cadherin re-expression, thus carcinoma cells revert into a noninvasive state in the absence of ongoing exposure to the micro-environmental signals. This plasticity might result in the formation of new tumor colonies of carcinoma cells exhibiting a histopathology similar to those of carcinoma cells in the primary tumor that did not undergo an EMT. It is likely that EMT is triggered by genetic and epigenetic alterations of the tumor cells and their interaction with the surrounding microenvironment including stromal cells and matrix components. Little is known on the mechanisms controlling the release of these EMT signals within a tumor. In part, the understanding of these mechanisms is complicated by the fact that the EMT signals controlling cell number and position within tissues are thought to be transmitted in a temporally and spatially regulated fashion from one cell to its neighbors. Such paracrine signaling is difficult to access experimentally [30].

## Conclusions

Our data demonstrate that Twist2 is up-regulated in breast carcinomas. Twist2 expression significantly increases and is correlated with tumor histological type and metastasis of breast cancer. Twist2 may be a potential diagnostic biomarker of breast carcinomas. The differential cellular distribution of Twist2 may be associated with its role in tumor progression. Our findings indicated heterogeneous expression of Twist2 in tumors may have a functional significance: the cytoplasmic Twist2 at tumor center and lymph metastases contributes to the maintenance of epithelial cancer characteristics with E-cadherin expression in a noninvasive state, while the nuclear Twist2 activates EMT transiently in the tumor invasion front to facilitate cancer cell invasion and metastasis.

## Materials and Methods

### Antibodies and Tumor Tissues

Anti-Twist2 monoclonal antibody was purchased from Abnova Biotechnology. Rabbit anti-Slug antibody was from Cell Signal Technology (CST, USA). Rabbit anti-erbB2 antibody was from Epitomics Inc. (USA). Rabbit anti-E-cadherin antibody was obtained from Santa Cruz Biotechnology (Santa Cruz, CA, USA). ABC Kits were purchased from Thermo Scentific, and DAB substrate kit from Pierce. The formalin-fixed and paraffin-embedded normal breast tissues and breast carcinomas were selected randomly from the tissue bank in the Department of Pathology, Zhongshan Hospital, Medical College of Xiamen University. The research protocol and design were approved by the Ethics Committee of Xiamen University (ID No: 20081106). The tissue microarray was purchased from Biomax Inc (USA). All of our clinical studies have been conducted according to the principles expressed in the Declaration of Helsinki.

### Cell Culture and Generation of Twist2-expressing Breast Cancer Cells

MCF-7 cell was obtained from the American Type Culture Collection (Manassas, VA, USA). The cells were cultured in DMEM medium supplemented with L-glutamine,10% FBS (Hyclone), and penicillin/streptomycin,and maintained in a humidified atmosphere of 5% CO2 at 37°C. The Flag-Twist2 (NM_057179) expressing plasmid and the pBabe-puromycin vector were co-transfected into MCF-7 cells using the lipofectamine2000^TM^ transfection reagent (Invitrogen) according to the manufacture's instruction. The Twist2 transient over-expressed cells and the vector control cells were collected after transfected for 48 hours. The Twist2-expressing stable clones and the vector control clones were obtained respectively through the selection with puromycin, and the Twist2 expression levels in the selected stable clones were then verified by immunoblot analysis with Twist2 and flag antibodies. And detected proliferation rate of transfected cells compared with vector control by viable cell counts using trypan-blue staining.

### Immunohistochemical Staining

Tumor classification and characterization of Twist2 expression was done on sections of formalin-fixed, paraffin-embedded samples of breast tissues. Sections were cut continuously and immunohistochemical staining of E-cadherin, ErbB2 and Slug were also performed on the adjacent sections as described previously [34]. Briefly, tissues were fixed in 10% buffered formalin and embedded in paraffin. Four-micrometer-thick sections were deparaffinized in xylene and rehydrated in graded alcohols and distilled water. After antigen retrieval, endogenous peroxidase activity was blocked with 0.3% hydrogen peroxide in methanol for 30 minutes followed by rehydration in PBS, and incubation with 5% goat serum for 60 minutes to bind nonspecific antigens. Sections were incubated overnight at 4°C with the primary antibodies. The immuno-signals were detected with the ABC Kits at room temperature. After rinsing, the sections were incubated with DAB, counterstained with hematoxylin, dehydrated, and then mounted. As a negative control, the primary antibody was replaced with normal mouse IgG. The sections were then analyzed by standard light microscopy.

### Confocal Immunofluorescent Staining

Twist2- and vector-transfected MCF7 cells were grown on sterilized cover slips for 20 hr, then fixed in 4% paraformaldehyde for 30 min at 4°C and permeabilized in 0.2% TritonX-100 in PBS at room temperature (RT) for 15 min. Then, cells were incubated with 2% BSA for 1 hr at RT to block nonspecific binding before the primary antibody reaction. Cover slips were incubated with the primary antibodies to Twist2 and E-cadherin overnight at 4°C, then washed with PBS and incubated with Alexa Flour 555, FITC-conjugated secondary antibody (Dako) for 1 hr in the dark at RT. Cover slips were washed three times in PBS, mounted with Vectashield (Vector Laboratories) containing 4, 6 diamidino-2- phenylindoledihydrocloride (DAPI) for DNA staining and analyzed using the laser scanning confocal microscopy (Olympus).

### Evaluation of Immunohistochemistry Results

Three independent observers who had no prior knowledge of the patient data reviewed the immunohistochemical sections. For staining results, all areas of each sample were examined and that with the greatest immunoreactivity was selected for quantification. For Twist2, cytoplasmic and nuclear immunoreactivity were determined. Positive cells showed brown granules in cytoplasm or cell nucleus [35]. Cytoplasmic staining was scored based on the percentage of positive tumor cells and staining intensity. The positive cell percentage was determined by calculating the percentage of positive tumor cells in total observed cells: 0, <10%; 1, 10%–20%; 2, >20%. The intensity was decided as: 0, no staining or ambiguous staining; 1, medium staining; 2, strong staining. The two scores were multiplied to evaluate staining: 0 to 1, negative; 2 to 4, positive (+). Nuclear staining was scored using a 400× magnification, and 100 nuclei were counted according to the percentage of nuclei showing positive immunoreactivity, which was graded on an arbitrary scale ranging from 0 to 2: 0, <10%; 1, 10%–20%; 2, >20%. A score of 2 was classified as high-level expression (+) and 0 to 1 as low-level expression (−). Membranous and cytoplasmic staining of E-cadherin was examined immunohistochemically using the same tissue as Twist2. Positive E-cadherin immunoreactivity was scored similarly to that of Imai T et al [36]. Weak or no expression of E-cadherin was regarded as aberrant E-cadherin expression, moderate to strong membranous and cytoplasmic staining of E-cadherin was classified as normal expression.

### Isolation of Subcellular Fractions, and Western Blot Analysis

Details of cell fractionation and Western blot analysis were performed as our previous publications [37], [38]. Protein Assay Kit for protein quantity analysis was purchased from Bio-Rad (CA,USA). The enhanced chemiluminescence (ECL) detection system was purchased from Amersham (IL, USA). All antibodies were described as materials.

### Statistical analysis

The Chi-square test and Fisher's exact test were used to assess differences. Spearman's rank correlation was used to determine whether there was a positive or negative correlation. P<0.05 was regarded as statistic significant. These analyses were performed using the SPSS 13.0 package.
